# In Memoriam—M. A. A. Wolff, M. D.

**Published:** 1892-01

**Authors:** 


### IN MEMORIAM— M. A. A. WOLFF, M. D.

Dr. M. A. A. Wolff, son of the grand rabbi of Copenhagen, died at midnight, Wednesday, September 30th, 1891, in the Homœopathic Hospital, at Kansas City, Mo., of vesico-rectal fistula complicated with stone in the bladder. He was for years a practicing physician, eminent in this school of medicine, and the author of several books in branches of which he made special study. He was a cultured man and a believer in the Jewish faith. A year and a half previously he was the subject of an unsuccessful operation in a St. Louis hospital.* He was brought to Kansas City for treatment, and for nearly half a year suffered excruciating agonies. Wednesday Rabbi Berkowitz spent much of the day at the dying man's bedside. The suffering of the man was so great that he was part of the time unconscious, but at one time when his mind was clear he told the rabbi that he was dying.

The Doctor's wife died years ago and he had no children. He was visited at the hospital by men of his faith, but they were strangers to him. On Friday, October 2d, after a simple service, he was buried with the rites of the Jewish faith.

---

*See THE HOMŒOPATHIC PHYSICIAN, 1890, pages 407, 501.

Dr. Wolff had been in this country several years. He spent some years in California, but of late called Gainesville, Texas, his home. He was sixty-three years old.

Dr. Wolff's father, whose ninetieth birthday was celebrated by all the nations of Europe last summer, is grand rabbi of Copenhagen. He is a man whose reputation is as wide as the world. At the time of his last birthday he was visited by the King of Denmark.

Dr. S. Mills Fowler, of Gainesville, Texas, writes of Dr. Wolff, "he was a skilled physician, a strict Hahnemannian, and an accurate and successful prescriber. He had been a great sufferer for years from cystic irritation and gravel and in his own person he had many times experienced the almost magical effects of the potentized simillimum."

---

